# The Arabidopsis Kinome: phylogeny and evolutionary insights into functional diversification

**DOI:** 10.1186/1471-2164-15-548

**Published:** 2014-07-01

**Authors:** Monika Zulawski, Gunnar Schulze, Rostyslav Braginets, Stefanie Hartmann, Waltraud X Schulze

**Affiliations:** Max Planck Institute of Molecular Plant Physiology, Am Mühlenberg 1, Golm, 14476 Germany; Bioinformatics, Institute of Biochemistry and Biology, University of Potsdam, Karl-Liebknecht-Str. 24-25, Golm, 14476 Germany; Department of Plant Systems Biology, University of Hohenheim, Garbenstraße 30, Stuttgart, 70599 Germany

## Abstract

**Background:**

Protein kinases constitute a particularly large protein family in *Arabidopsis* with important functions in cellular signal transduction networks. At the same time *Arabidopsis* is a model plant with high frequencies of gene duplications. Here, we have conducted a systematic analysis of the *Arabidopsis* kinase complement, the kinome, with particular focus on gene duplication events. We matched *Arabidopsis* proteins to a Hidden-Markov Model of eukaryotic kinases and computed a phylogeny of 942 *Arabidopsis* protein kinase domains and mapped their origin by gene duplication.

**Results:**

The phylogeny showed two major clades of receptor kinases and soluble kinases, each of which was divided into functional subclades. Based on this phylogeny, association of yet uncharacterized kinases to families was possible which extended functional annotation of unknowns. Classification of gene duplications within these protein kinases revealed that representatives of cytosolic subfamilies showed a tendency to maintain segmentally duplicated genes, while some subfamilies of the receptor kinases were enriched for tandem duplicates. Although functional diversification is observed throughout most subfamilies, some instances of functional conservation among genes transposed from the same ancestor were observed. In general, a significant enrichment of essential genes was found among genes encoding for protein kinases.

**Conclusions:**

The inferred phylogeny allowed classification and annotation of yet uncharacterized kinases. The prediction and analysis of syntenic blocks and duplication events within gene families of interest can be used to link functional biology to insights from an evolutionary viewpoint. The approach undertaken here can be applied to any gene family in any organism with an annotated genome.

**Electronic supplementary material:**

The online version of this article (doi:10.1186/1471-2164-15-548) contains supplementary material, which is available to authorized users.

## Background

Protein kinases constitute a protein family with functions in cellular signal transduction pathways. In the model plant *Arabidopsis thaliana*, about 4% of the genes encode protein kinases, which can be referred to collectively as the kinome [1]. These different protein kinases can be subdivided into several families according to their function, structure, and phylogenetic relationships. Roughly 60% of all protein kinases belong to the large superfamily of receptor kinases (RLK), including the large family of transmembrane leucine-rich-repeat (LRR) receptor kinases. Also the so-called receptor-like cytoplasmic kinases (RLCK), which lack extracellular and trans-membrane domains are part of the receptor kinase clade. The clade of soluble kinases consists of the most prominent families, namely the cyclin-dependent kinases (CDK) involved in cell-cyle regulation, the mitogen-activated protein kinases (MAPK, MAPKK, MAPKKK), which constitute transmission cascades for responses to extracellular stimuli, the AGC kinases, and the kinases decoding calcium signals (CDPK-SnRK superfamily). Although mechanisms leading to expansion of the receptor-like kinases in *Arabidopsis* were suggested [2], a comprehensive analysis of the entire *Arabidopsis* kinome with respect to gene duplication patterns has not been carried out so far.

Gene duplication events present an important mechanism for the generation of evolutionary novelties [3], and at least six different types of duplications including tandem duplications, whole-genome and segmental duplications, as well as transpositions can be distinguished [4]. For example, duplication events may affect local genes by single-gene duplications, blocks of genes on chromosomes by segmental duplications, or entire genomes through whole-genome duplications. Each mechanism of duplication results in potentially changed expression context of a gene or leads to genetic and functional redundancies. However, due to the reduction of selective pressure on redundant gene copies in duplicated regions, duplicates may be lost or pseudogenized. The term ’syntenic region’ is widely used in the context of gene duplication analysis and evolutionary history of genes and genomes. In classical genetics, synteny refers to the colocalization of genes or genomic regions on the same chromosome [5]. The concept of collinearity on the other hand refers to a conserved gene order between the ancestral and the potentially duplicated genomic region and may thus be used to infer synteny in the context of gene duplications [5–7].

Several models for the retention and loss of duplicated genes have been proposed and it is believed that at least in plants, genes are retained or lost in a biased manner with respect to their mode of duplication and functional context [8–10]. Plants, especially angiosperms, are known for their high frequency of chromosomal and whole-genome duplications. *Arabidopsis thaliana* alone has experienced at least two recent whole genome duplication events in the period between its divergence from *Carica papaya* (∼72 million years ago) and *Arabidopsis lyrata* (∼10 million years ago) commonly referred to as α and β duplication events, respectively. In addition, there was an ancient paleohexaploidy event shared between all rosids [6]. As a consequence, the expansion and functional diversification of gene families was largely shaped by gene duplication events and a number of studies have reported their impact on the evolution of resistance genes [11] and various other large gene families [6, 9].

In this study, the freely available MCScanX toolkit [7] was used to detect collinear regions in *Arabidopsis thaliana* and classify duplicated kinase genes according to their most likely mode of generation. Classifications were further refined by the MCScanX-transposed extension using *Arabidopsis lyrata* and *Populus trichocarpa* as outgroups. To gain insight into patterns of retention and loss of duplications within protein kinase families, inferred syntenic regions were mapped onto a phylogeny of 940 kinases and then linked to gene expression data, family gene annotations and loss-of-function phenotypes. Besides assembly and phylogenetic evaluation of the *Arabidopsis* kinome, our study provides insights into the functional diversification among the protein kinases in the context of gene duplications.

## Methods

### Phylogeny of Arabidopsis kinases

An alignment of 491 eukaryotic protein kinases was downloaded on Feb 9, 2012 from http://kinase.com/human/kinome/phylogeny.html, and this alignment was used to compute a profile Hidden Markov Model (HMM) using the software HMMer [12]. All representative gene models from *Arabidopsis thaliana* (TAIR10_pep) were searched against the profile HMM using HMMer. In total, 1,045 sequences generated hits with an E-value lower than 0.01. These sequences were then aligned to the profile HMM. Two sequences (AT1G11300.1 and AT2G32800.1) each had two distinct kinase domains and both domains per gene were therefore included as separate sequences in the alignment, resulting in an alignment of 1,047 distinct Arabidopsis kinase domains (Additional file 1). All alignment positions not part of the profile HMM were removed from the alignment. In addition, all sequences covering less than 70% of the profile HMM were removed using the software REAP [13]. The cutoff value of 70% corresponded to a threshold value in sequence coverage distribution with sharp decline of sequence coverage for 111 kinase domains below coverage of 70% (Additional file 2). The final alignment then consisted of 317 columns from 942 sequences (kinase domains) and was used to compute a maximum likelihood phylogeny and 100 bootstrap replicates using the PROTCATWAG model of the RAxML program [14].

The mapping of genes to kinase families was based on an extensive literature search [15]. In the case of unkown/unreported family annotation, the gene phylogeny as well as domain structural information was used to infer the most likely annotation for genes according to their clade membership. This approach resulted in the (re)assignment of 115 previously lacking or ambiguous annotations. The original three file of the phylogeny has been deposited at Dryad under the reference number pq7d7 (doi:10.5061/dryad.pq7d7).

### Detection of syntenic blocks and classification of duplication types

To predict segmentally duplicated blocks in the *Arabidopsis thaliana* genome and further classify and count other types of gene duplications, a local installation of the MCScanX toolkit was obtained from the MCScan webpage (http://chibba.pgml.uga.edu/mcscan2/). To prepare sequences for analysis, a local installation of the BLAST + suite (version 2.2.27) was obtained from NCBI. Protein sequences of representative gene models and associated annotation files were downloaded from TAIR v10 (ftp://ftp.arabidopsis.org/home/tair/Genes/TAIR10_genome_release/) for *Arabidopsis thaliana* and from phytozome.net (http://www.phytozome.net/) for *Arabidopsis lyrata* [16] and *Populus trichocarpa* [17]. *Arabidopsis thaliana* whole genome protein sequences were queried against databases of *Arabidopsis thaliana*, *Arabidopsis lyrata* and *Populus trichocarpa*, using blastp with an E-value cutoff of 10^-5^ and restricting the output to a maximum of five hits per gene to serve as input for the MCScanX toolkit, which was used to detect and classify syntenic regions. Detection of collinear blocks and duplication classification were performed by the MCScanX algorithm and associated downstream tools using default parameters. To further enhance the duplication classification and allow for the detection of transposed genes, the MCScanX-transposed extension was employed using *Arabidopsis lyrata* and *Populustrichocarpa* as outgroups.

### Enrichment analysis and calculation of expected counts

For each kinase family the ratio of expected to observed counts per duplication event was calculated. This ratio for tandem duplications was plotted against the ratio for segmental duplications in a bidirectional boxplot [9]. The expected duplication frequency of segmental and tandem duplication events in each family was calculated as follows: For tandem counts, a simulation was carried out, placing N genes of size 1 kb (where N is the size of the corresponding gene family) in a genome of approximately 100000 kb and counting how many pairs of genes were within a 50 kb window. The gene family size (N) was varied between 10 and 300 in steps of 10. Each simulation was repeated 1000 times, and the results were averaged to yield the expected tandem counts for each size class. Relying on previous reports on the frequency of segmental duplications in *Arabidopsis thaliana* [18], the expected proportion of the genome present in at least one segmentally duplicated block was approximately 75%. Thus, assuming no bias, the average count of segmentally duplicated genes in each gene family can be estimated by the relation segexp = N * 0.75, where N is the number of genes in the respective gene family.

To evaluate significant differences in duplication types between families, an enrichment analysis was carried out by employing Fisher’s exact test under the null hypothesis of no association between a particular subfamily and frequency of a particular duplication mechanism. Each combination of subfamily and duplication mechanism was tested separately, and the obtained p-values were corrected for multiple hypotheses testing by Benjamini-Hochberg correction [19]. Additionally, Pearson residuals from Chi-squared tests were used to assess the direction (enrichment/depletion) and strength of deviation from associations between sub-family and duplication mechanism expected under the null hypothesis.

### Data analysis and visualization

Visualization of phylogenetic trees, simulations and statistical analyses were conducted in R (http://www.r-project.org/) using packages ape [20] and phangorn [21]. Customized Perl scripts were used to parse input and output files to and from the MCScanX-utility. Results were stored and queried using the R-package RSQLite (http://cran.r-project.org/web/packages/RSQLite/index.html) in combination with a SQLite3 database which is available on request. Interaction networks were visualized using Cytoscape version 3.0.2. [22]. Phylogenetic trees were computed with Raxml, and visualized with the program FigTree (Version 4.1, A. Rambaut; http://tree.bio.ed.ac.uk/software/figtree/). Gene expression samples from various developmental stages and tissues specific to the set of investigated kinases were downloaded from Genevestigator (https://www.genevestigator.com/gv/).

### Public data sets

Subcellular locations were used based on the consensus location in SUBA3 [23]. Phenotypes of loss-of-function mutants were obtained from [24]. Protein-protein interaction data were obtained from AI1 [25]. Information on myristoylation [26], phosphorylation [27] and functional annotation [28] was taken from supplementary materials of mentioned publications and/or from TAIR [29].

## Results

### Phylogeny of the Arabidopsis kinome

**Figure 1:**
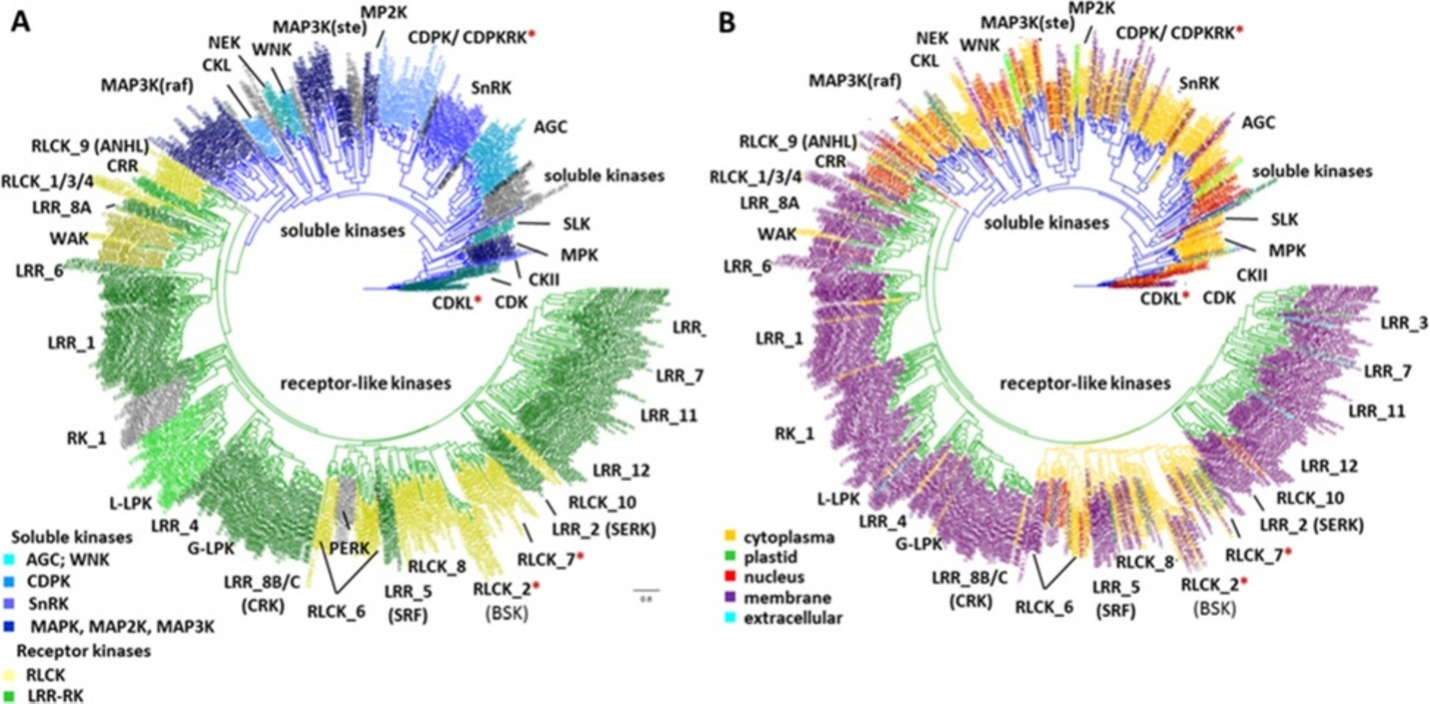
**Maximum Likelihood phylogeny of 942 kinase domains in** ***Arabidopsis thaliana.*** **(A)** Color coding according to functional families separating soluble kinases (blue) from receptor kinases (green). **(B)** Color coding according to subcellular location from SUBA3.

For a comprehensive analysis of the *Arabidopsis* kinome, we carried out a phylogenetic analysis based on the kinase domains of kinase-domain containing proteins. Proteins were defined as “kinase” based on significant match to a Hidden Markov Model (HMM) generated from an alignment of eukaryotic kinases (kinase.com). This resulted in a phylogeny of 942 protein kinase domains of 940 proteins (Figure 1), comprising 3.4% of the 27416 representative gene models in *Arabidopsis* based on the TAIR10 genome annotation. Atypical protein kinases of the plastid [30] and other (mitochondrial) atypical kinases such as PDK (AT3G06483), a kinase specifically involved in phosphorylation of the E1α subunit of the pyruvate dehydrogenase complex [31], were not part of our analysis. Histidine receptor kinases as members of two-component signalling [32] were also not included here. Our phylogeny is consistent with early postulations of about 1,000 protein kinases in *Arabidopsis* [1] after the publication of the *Arabidopsis thaliana* genome [33].

The phylogeny of *Arabidopsis* kinases showed a clear division into two major clades of 561 membrane-located receptor kinases and 381 soluble kinases (Figure 1A). The clade of soluble kinases consisted of 21 distinct published kinase families and most functionally characterized subfamilies form separate clades. These included four kinase families of the mitogen-activated kinase cascades (MAPK, MAP2K, raf-like and ste-like MAP3K), which are known for transmission of various responses to changes in gene expression [34]. Kinases annotated as MAP3 kinases were split into two separate clades as already noted earlier [35]: the 37 members of the ste-like MAP3 kinases grouped together with MAP2 kinases, and the 48 members of raf-like MAP3 kinases formed a separate clade. To date, few phosphorylation targets for these different MAP3 kinases are known [15]. For the ste-like MAP3 kinases several MAP2 kinases were found among the target proteins, confirming the classic cascade model in which ste-like MAP3 kinases phosphorylate and activate MAP2 kinases as well as transcription factors [36, 37]. In contrast, raf-like MAP3 kinases contain well known kinases like VIK1 (At1g14000) [38], STY-kinases (At2g17770, At4g35780, At4g38470) and CTR1 (At5g03730). VIK1 was found to be involved in regulation of tonoplast transporters [38], and CTR1 interacts with the Ethylene receptor ETR1 (At1g66340) and phosphorylates the transcription factor EIN3 (At3g20770) [39]. Since so far no MAP2 kinases were found to be phosphorylated by raf-like MAP3 kinases, it is most likely that they are a mis-annotated kinase family and do not actually function in MAPK-signaling.

Kinases decoding calcium signals were grouped into the families of calcium-dependent kinases (CDPK) and CBL-interacting kinases (CIPK/SnRK3). The latter group was located on the same clade as two other groups of Snf-related kinase (SnRK) [40]. The AGC kinases act as effectors of second messengers, are involved in many different processes from blue light perception to auxin signalling and, as expected, form a distinct family [41]. Another soluble kinase family involved in hormone signalling and represented as a separate clade in the phylogeny is the family of Shaggy-like kinases (SLK/GSK3) [42]. These kinases act as signal transducers from plasma-membrane located processes to transcription factors or other kinases, and they are best characterized in the context of brassinosteroid signalling [43]. Kinases involved in the regulation of cell organisation and cell division group into the families of cyclin dependent kinases (CDK) [44], the casein and casein-related kinases (CKII and CKL) [45, 46], the never-in-mitosis kinases (NIMA/NEK) [47] as well as the three AURORA kinases [48]. With-no-lysine kinases (WNK) contribute to the regulation of circadian rhythm [49].

In addition, 48 soluble kinases without known family annotation were found to form separated clades in the phylogenetic tree, but for most of these kinases no functional information is available yet. Based on their placement on the phylogeny, we were able to annotate 20 of these soluble kinases: one was defined as AGC kinase (PDK1;3, AT2G20050), one as CDK (CDKC1;2, AT3G01085), three as members of the Raf-like MAP3 kinases family and 15 as ste-like MAP3 kinases. All newly identified kinases were marked with one asterisk in the proposed annotation in Additional file 1.

The annotation of receptor-like kinases on the phylogeny confirmed earlier efforts of receptor kinase classification based on whole sequences and extracelluar domain structures [50]. The division of this large clade into receptor kinases (RLK) and receptor-like cytoplasmatic kinases (RLCK) corresponds well with the information of subcellular location obtained from SUBA3 [23]. Sequences without annotation were assigned based on their sister-group relationship to known kinases (Additional file 3). These newly annotated receptor kinases were assigned with one asterisk in Additional file 1. In those cases where existing protein annotations were in disagreement with the annotation of the majority of kinases in the same clade, these kinases were renamed and marked with two asterisks in Additional file 1 (see also Additional file 3). Branches in the phylogeny with not-annotated or mis-annotated kinases did not differ in their bootstrap values from the already annotated kinases. Thus, the newly annotated kinases had the same degree of support for family membership from the bootstrap values as already annotated family members.

In contrast to the family annotation for soluble kinases, the functional context of only few RLKs is known. Therefore, the functional annotation of RLKs is mainly derived from domain structure and homologies to kinases in yeast or animals instead of activating substrate or acting pathway. Further investigation of biological processes and targets for most of the RLKs is needed to provide a similar quality of functional annotation for the RLK families as is already available for the soluble kinase families.

Subcellular localizations for the soluble kinases according to SUBA3 [23] ranged from nucleus to plasma membrane (Figure 1B) and is in good agreement with the division of the phylogeny into the cytoplasmic kinases, receptor kinases and membrane-located cytoplasmic kinases. Within the soluble kinase clade, membrane location was often achieved by posttranslational modifications. Myristoylation is a posttranslational modification of proteins allowing a reversible protein association with plasma membrane [51]. Currently, 437 proteins are known to be myristoylated (Additional file 1), among them 83 kinases from our analysis [26]. The subfamilies RLCK_2 and RLCK_7 contained membrane associated kinases with 10 and 12 members known to be myristoylated. Also most calcium-dependent kinases, all CDPK-related kinases, and all CDK-like kinases are soluble kinases with known reversible membrane interactions.

### The origin of the Arabidopsis kinome: insights from gene duplication analysis

**Figure 2:**
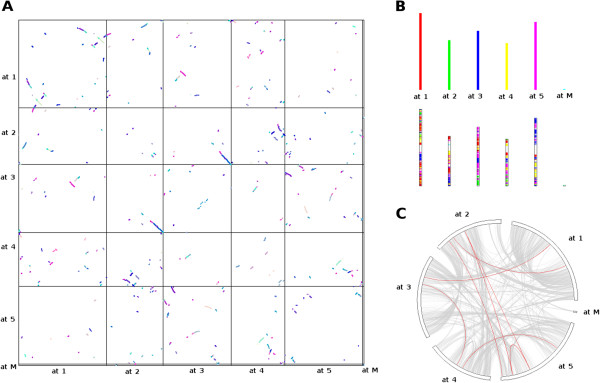
**Visualization of results as provided by the MCScanX utility. (A)** Dot plot indicating segmentally duplicated regions in the *Arabidopsis thaliana* genome. The axes indicate the genes on the five chromosomes (at1 to at5) and mitochondria (M). Colored dots denote different duplication events. **(B)** Bar plot showing the estimated proportion of segmental duplications on each chromosome. The corresponding origin for each chromosome is indicated in color. **(C)** Circle plot of kinase family-specific collinear regions (red curves) between chromosomes against the background of collinear regions in other genes (grey).

Whole-genome comparisons of *Arabidopsis thaliana* against itself as well as against *Arabidopsis lyrata* and *Populus trichocarpa* were carried out using protein Blast [52], and results were used as input for the MCScanX and MCScanX-transposed utilities. By comparing the *Arabidopsis thaliana* genome against itself, 7496 (26.05%) genes in 224 collinear segments were identified to be the result of at least one whole-genome or segmental duplication event (Figure 2A). The median and maximum inferred duplication depth was 1 and 7, accounting for 9 and 14646 genes (51%), respectively. In total, about 82% of all genes in *Arabidopsis* were inferred to result from any type of duplication event (Table 1). All five chromosomes as well as the mitochondrial genome were subjected to substantial duplication events (Figure 2B). Among the duplicated genes, 335 genes (4.5%) had a kinase family annotation, and duplication events affecting kinases were also distributed across all five chromosomes (Figure 2C).

**Table 1:** **Summary of duplications identified by MCScanX (** ***Arabidopsis thaliana*** **against itself)**

Duplication mechanism	All genes (%)	Kinase-specific (%)
WGD/segmental	7496 (26.1)	335 (35.7)
Dispersed/transposed	11779 (41.0)	444 (47.4)
Proximal	1323 (4.6)	67 (7.2)
Tandem	2875 (10.0)	89 (9.5)
Singleton	5233 (18.2)	2 (0.2)

For each duplication mechanism and singleton genes the number and percentage of occurrences is listed in the set of all genes and kinase encoding genes.

### Distribution of duplication patterns to kinase subfamilies

**Figure 3:**
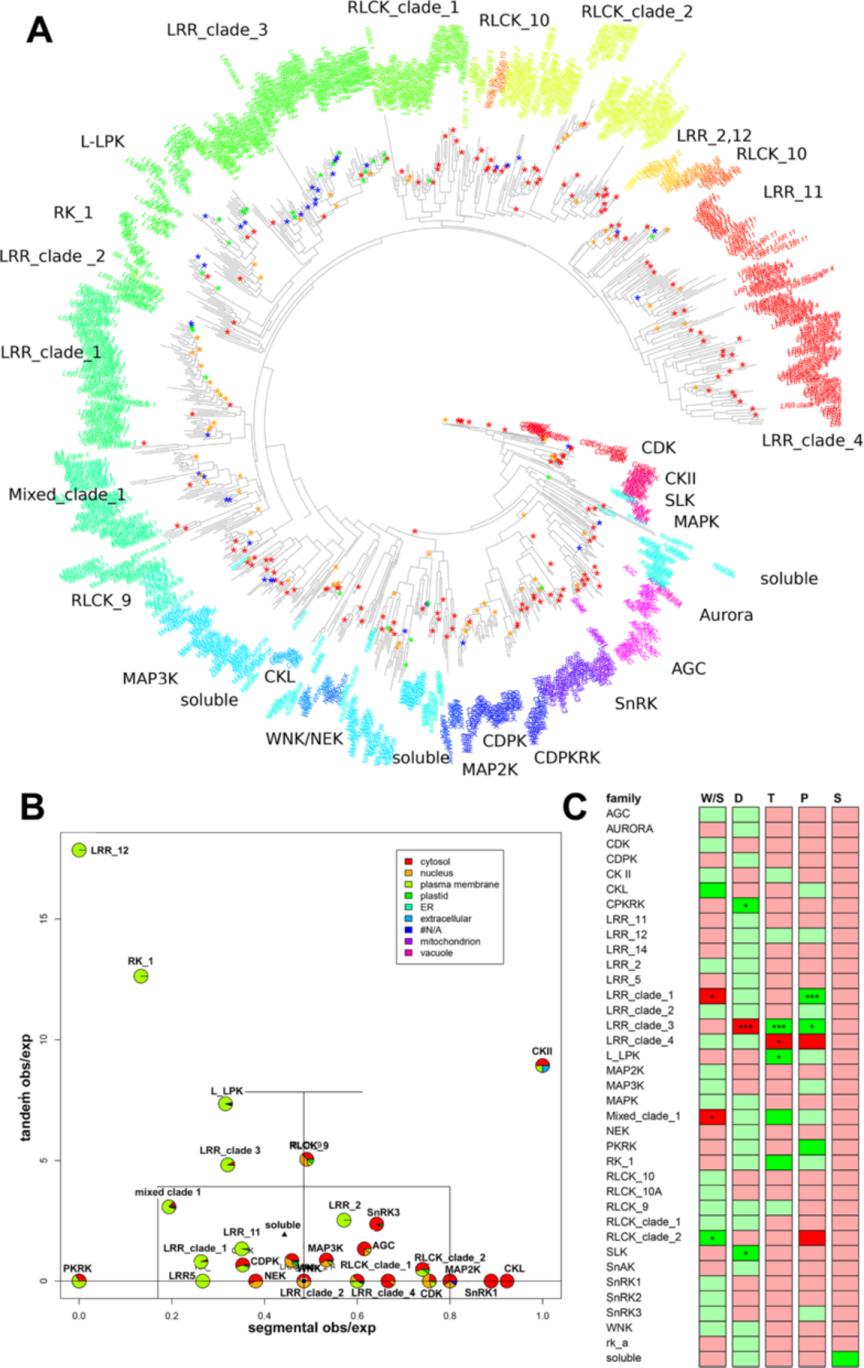
**Duplication events in** ***Arabidopsis*** **kinases. (A)** Phylogenetic tree of kinase families indicating gene duplication events**.** Small subfamilies were collapsed into artificial monophyletic clades as discussed earlier and indicated in the figure. Duplications are marked by asterisks at branching points corresponding to the most recent common ancestor of duplicates. The different types of duplication events are color-coded. red: segmental, blue: tandem, green: proximal, and orange: transposed. **(B)** Box plot of ratios of observed to expected tandem and segmental duplication frequencies for each family. The boxed region is centred on the median ratios (indicated by a black square) for tandem and segmental duplications. The mean of ratios is marked by a black triangle. The boxed region and additional lines refer to one and two standard deviations above and below the median ratios. Pie charts for each family indicate the localization of genes according to SUBA3 as the proportion of all genes in that family. **(C)** Results of the enrichment analysis. For each family and duplication type, the Pearson residuals from Chi-square tests are plotted together with the corresponding significance levels from Fisher’s exact tests. Duplication types are coded as W/D (whole-genome or segmental duplication), D (dispersed), T (tandem), P (proximal) and S (singleton). Red and green color gradients correspond to negative and positive Pearson residuals indicating depleted and enriched counts for each type of duplication. The significance is coded as follows: *corresponds to p < 0.05, ***corresponds to p < 0.001. P-values were adjusted for multiple testing by applying the Benjamini-Hochberg correction.

To better visualize the relationship between duplication events and kinase family evolution, the segmental, tandem, proximal and transposed duplicate pairs inferred by the MCScanX-transposed extension were mapped onto the kinase phylogeny (Figure 3A). Disregarding the exact genetic mechanism, the frequency of tandem duplications within a gene family and genome of given size was estimated by counting the number of gene pairs within a critical distance (50 kb windows) assuming a random spatial distribution of genes in the genome. Clearly, in larger gene families, more tandem duplications could in principle be observed by chance, resulting in a higher expected value. Due to the fixed limit of genome size, the expected frequency of tandem duplications grows nonlinearly with increasing family size. For segmental duplications, about 75% of the *Arabidopsis* genome is suggested to be present in at least one segmentally duplicated block [9]. Thus, assuming unbiased retention of segmentally duplicated genes, each family should contain a proportion of about 75% segmentally duplicated genes. Proximal duplications were defined as gene pairs being separated on the same chromosome by more than 19 other genes. Information gained from the analysis of segmental and tandem duplication frequencies in each kinase subfamily was summarized in a bi-direction boxplot. Applying this approach to the kinase-specific dataset, the ratio of observed to expected duplications was plotted as a bi-directional box-plot (Figure 3B). The subfamilies CKII, RLCK 10A, and CKL showed an increased observed to expected ratio for segmental duplications (> +1SD above the median). In contrast the family LRR_12 and RK_1 can be considered as a kinase families with increased observed to expected ratio for tandem duplications (> +2SD above the median) and decreased ratios for segmental duplications (< -1SD), respectively. These results are summarized in Additional file 4.

Pie charts in Figure 3B indicate the proportion of genes that are assigned to particular subcellular compartments. Some kinase families, such as RLCK_9 contained genes with very different consensus localizations [23]. Treating the majority of subcellular localization within each subfamily as representative, we observed a tendency of receptor kinase families with predominant plasma membrane localization to preserve genes arising from tandem duplications rather than those originating from segmental duplications. Conversely, most cytosolic kinase subfamilies exhibit moderate to low ratios of observed to expected counts for tandem duplications and relatively large ratios for segmental duplications. With the exception of CKII, soluble kinase subfamilies show increased ratios in either tandem or segmental duplications but not in both. Tandem duplicates were already proposed as one of the major mechanisms of expansion of the large receptor kinase family in *Arabidopsis* [2]. Although tandem duplication events are not predicted to be the major mode of gene copy generation for the entire set of kinase families in this study (Table 1), a certain tendency to retain tandem duplicates within the receptor kinases could be confirmed.

An enrichment analysis of particular duplication events associated with specific kinase subfamilies was performed by Fisher’s Exact test (Figure 3B) and obtained p-values were corrected for multiple testing [19]. The LRR clade 1 (containing the subfamilies LRR_6A, LRR_6B, LRR_1), and the mixed clade 1 (containing the subfamilies CRR, RLCK_1, LRR_8A, RLCK_3, RLCK_4, WAK), were found significantly depleted (p < 0.05) for WGD/segmental duplications, while the LRR clade 2 (containing the subfamilies LRR_9A, LRR_9) was significantly enriched (p < 0.05) for duplicates originating from segmental duplications. LRR clade 3 (containing the subfamilies LRR_4, LRR_8B, LRR_8C) was found to be depleted for dispersed duplicates (p < 0.001) and enriched for tandem duplicates (p < 0.001) and proximal gene copies (p < 0.05). LRR clade 1 and LRR clade 3 were also significantly enriched proximal duplication (p < 0.001 and p < 0.05, respectively). All subfamilies, except the non-characterized soluble kinase subfamily, were found to be weakly depleted for singleton genes.

### Functional divergence of duplications

**Figure 4:**
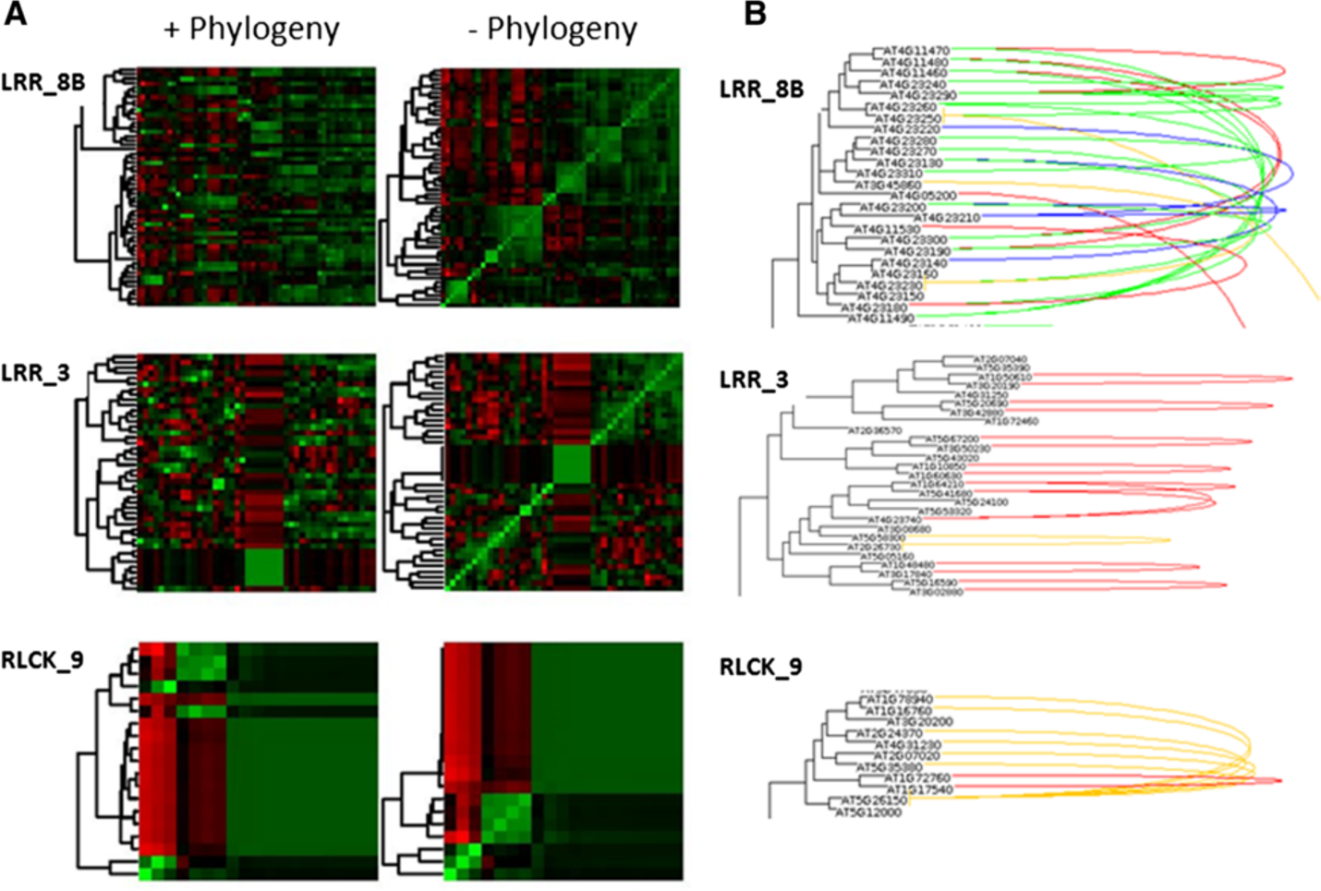
**Relationship between phylogeny and gene coexpression patterns. (A)** Coexpression analysis for subfamilies LRR_8B, LRR_3, and LRCK_9. Phylogenetic information was incorporated by reordering the rows according to the topology in the phylogenetic tree (left) or disregarded (right). Green color indicates high coexpression values, and red color indicates dissimilarity in expression context. Square patches of similar color denote agreements between the clustering of columns and rows. **(B)** Phylogenetic relationship and duplication annotation correspond for members of the LRR_8B, LRR_3, and LRCK_9 subfamily. Genes are connected by curves if they are identified as duplicated pairs by proximal duplication (green), segmental duplications (red) and transpositions (orange). In the case of transposition events, the ancestral gene is indicated by a vertical line.

To assess the correlation between different duplication mechanisms and the functional divergence among duplicated genes within kinase families, gene expression sets from various developmental stages and tissues were obtained from Genevestigator (https://www.genevestigator.com/gv/) and the coexpression of genes was estimated by Pearson correlation for all pairs of genes in the kinase phylogeny. The coexpression values were organized as heat maps in which the ordering of the columns corresponded to hierarchical clustering of genes according to their coexpression and the rows were reordered according to the phylogeny and phylogenetic topology for all kinase subfamilies (Additional file 5). In general, ordering of the rows based on phylogeny disrupted clusters of coexpressed genes.For most kinase families, co-expression clusters were lost once phylogenetic information was included in the expression heat map (e.g. LRR_8B family, Figure 4A). However, in some cases, the heat maps revealed interesting patterns of preservation of coexpression clusters within phylogenetic groups. For example, expression patterns corresponding to the LRR_3 subfamily revealed conservation of expression patterns also when rows were ordered according to the phylogenetic topology. For the RLCK_9 subfamily, we observed duplicated genes with conserved as well as diverse expression. In the cluster of LRR_8B subfamily there were high frequencies of proximal duplications, while coexpressed LRR_3 genes particularly originated from segmental duplication events. In the RLCK_9 family we observed a high degree of transposed duplications originating from the same ancestral gene (Figure 4B). The coexpression analysis suggests that proximal duplications were particularly associated with functional diversification, and segmental duplication events showed a tendency for conservation of expression patterns.

To assess the significance of protein kinases functional diversification in context of whole plant functions, we studied the distribution of phenotypes from loss-of-function kinase mutants [24]. Currently, about 2,400 genes with detectable loss-of-function phenotypes in *Arabidopsis* were identified, among them 76 kinases represented in our phylogeny. Thus, kinases constitute 3.25% of all genes with detectable loss-of-function phenotype. Overall, 45% of these kinases show a conditional phenotype, 29% a morphological 18% a lethal, and 8% a biochemical phenotype (Additional file 6). Most of these phenotypes were described for soluble kinases (soluble kinases: 11.5%; receptor kinases: 5.7%), particularly calcium-dependent kinases, all three SnRK-families, AGC kinases as well as six of 48 kinases without family annotation. The loss-of-function mutants in kinases were generally significantly enriched for essential phenotypes (p = 0.020, Fisher’s Exact Test), cellular and biochemical phenotypes (p < 0.001, Fisher’s Exact Test) as well as for conditional phenotypes (p = 0.002, Fisher’s Exact Test) compared to loss-of-function mutants in non-kinase genes. There was no enrichment for morphological phenotypes among the kinase mutants. Kinases with essential phenotypes were observed to contain a high proportion of dispersed duplications (p = 0.031), while kinases resulting in morphological loss-of-function phenotypes were particularly enriched for segmental duplications (p = 6.48E^-95^), supporting the tendency for functional diversification for dispersed duplications and functional conservation in segmental duplications.

### Kinases within the cellular interaction network

**Figure 5:**
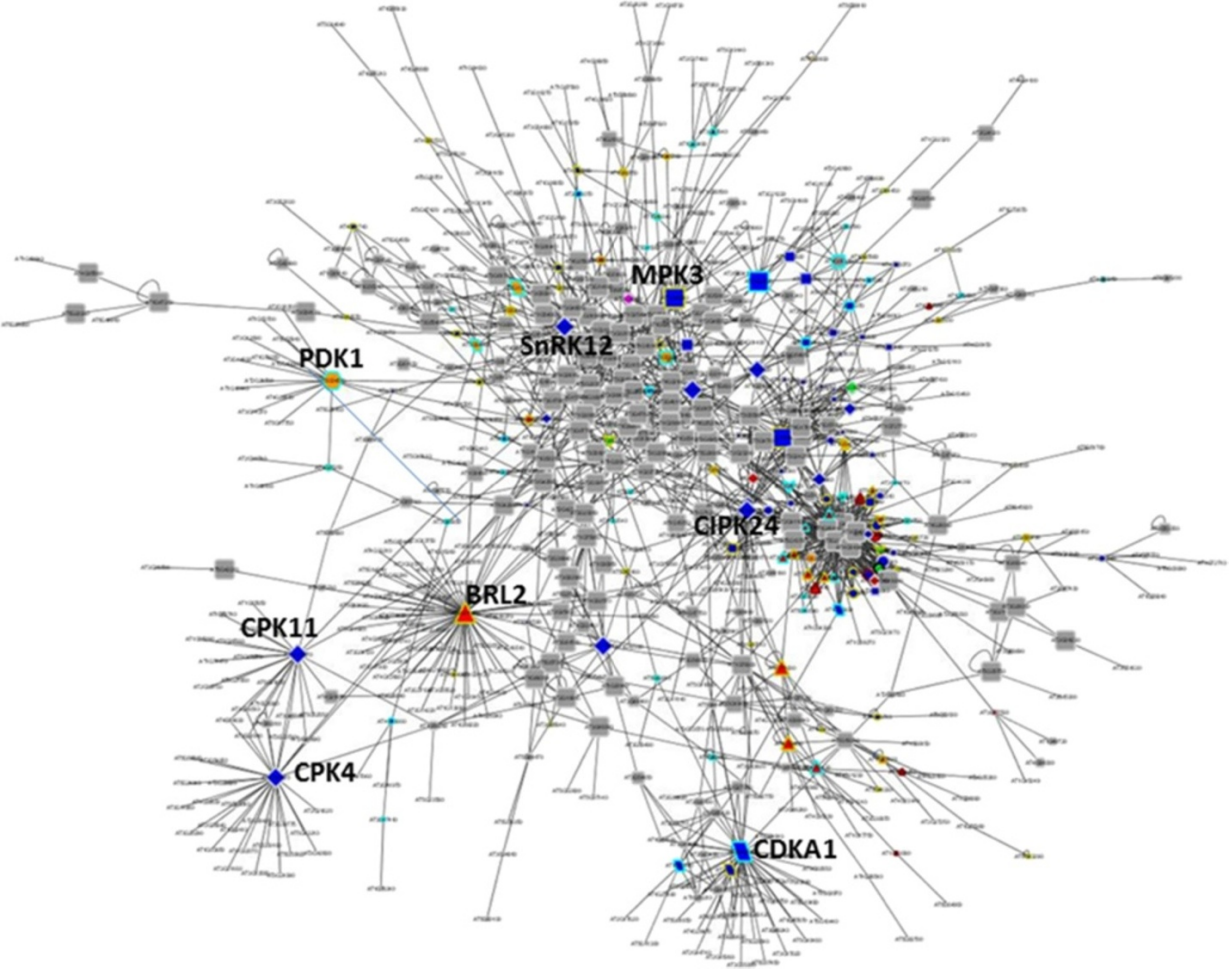
**Interaction network of protein kinases and their first interaction neighbours based in AI1. Node shape**: Square: MAP-Kinases including MAP2K and MAP3K; Hexagon: AGC kinases; Parallelogram: CDK; Diamond: CDPK and SnRK; Triangle: Receptor kinases; Vee: Receptor-like cytoplasmic kinases (RLCK); Circle: other soluble kinases. **Node colors** indicate subcellular location of kinases according to SUBA3: blue = cytosol; pink = endoplasmic reticulum; purple = extracellular; yellow = mitochondrion; orange = nucleus; green = plastid; red = plasma membrane. **Node border color** indicates duplication type: cyan = WGD/segmental; beige = dispersed; dark green = proximal; black = singleton; dark red = tandem duplications.

For 243 kinases analysed here, interaction partners are known from the *Arabidopsis* interactome AI1 [25]. In this network, some MAP-kinases, some members of the RLK superfamily and the AGC-kinase PDK1 (AT5G04510) had a particularly large number of interaction partners. The average degree (number of interactions) in this network of all protein kinases was 4.7. The protein kinases with degree higher than 20, thus having 20 or more interaction partners, were receptor kinase BRL2 (AT2G10950) with degree 64, followed by CDKA;1 (AT3G48750) with degree 43 and kinases CPK4 (AT4G09570), CIPK24 (AT5G35410), SnRK1.2 (AT3G29160), CPK11 (AT1G35670) and MAP-kinase MPK3 (AT3G45640) with degrees of 33, 33, 29, 24, and 20 (Figure 5). These kinases clearly were hub proteins with high degree separating and connecting individual functional subnetworks. For example, BRL2 interacted with a high number of proteins involved in hormone metabolism, CIPK24 had a high number of interactors with transport functions, while transcription factors or proteins of nutrient and sugar physiology were over-represented among the interactors of MPK3 and SnRK1.2, respectively. In general, most of these hub kinases displayed a conditional loss-of-function phenotype (Additional file 6), with exception of CDKA1 which has an essential phenotype, and BRL2 with a morphological phenotype. Furthermore, most of the hub kinases were not derived from duplication events, with the exception of BRL2 and MPK3 (dispersed duplication) and CDK (segmental duplication). Degree distribution for the protein kinases was highest for singletons (7) and for kinases without assigned genome duplications (5.3), and it was found lower than average for proteins with tandem (3.5) and proximal duplications (1.5). In yeast, there were early notions that network hubs showed a tendency for accumulation of essential phenotypes [53], but this finding was blurred with emergence of larger data sets [54]. In the whole *Arabidopsis* genome, although single gene copies had a tendency for more frequently displaying an essential phenotype, there was no general relationship between single copy genes and high degree in interaction networks [24]. However, this analysis was never broken down to functional categories. Although kinases were in fact under-represented among all the single copy genes in Arabidopsis [55], in our study these seemed to be of central role in the plant interaction network.

## Discussion

This study aimed at investigating the role of gene duplication events in the serine-threonine-tyrosine kinase complement of *Arabidopsis thaliana*. We constructed a phylogeny of eukaryotic kinase families and undertook efforts to link gene duplication events to functional diversification or conservation based on gene expression data.

### Kinome phylogeny

The phylogeny of *Arabidopsis* kinase subfamilies has been intensely studied for individual kinase families, especially for CDPKs [40], MAP-Kinases [56], or lectin receptor kinases [57], and members of the large family of receptor kinases [58]. Recent genome wide and species wide approaches in classification and annotation of eukaryotic protein kinases were based on Hidden Markov Model profiles [59, 60]. For plants, the most comprehensive classification can be found in the Eukaryotic Kinase and Phosphatase Database (EKPD) [59]. Overall, in our study a total of 111 *Arabidopsis* kinases listed in EKPD were not part of our phylogeny (Additional file 7). In contrast to EKPD, here kinase domains we also filtered for a minimum of 70% sequence coverage of the HMM representing the kinase domain, assuming that kinases with less than 70% coverage of the whole HMM kinase domain would not function as kinases. 94 proteins with an annotated kinase domain were excluded on this basis. Their kinase domains showed large gaps in the sequence covering conserved regions of the model kinase domain, with occasionally even half of the domain missing. Thus, especially the 50 excluded members of the receptor kinase group may have functionally degenerated kinase domains. In particular, 26 members of the atypical kinases were excluded, which show high similarities to prokaryotic kinases and are particularly abundant in plastid and mitochondrial location [30]. Another 17 kinases listed in EKPD did not match the HMM profile we used as template. Two plastidial yet uncharacterized kinases matching our HMM profile of an eukaryotic kinase were not classified as kinase in EKPD. Therefore, family-specific HMM models, as already used in EKPD, will be valuable in annotation of all kinases in Arabidopsis and eukaryotes in general. Histidine-receptor kinases [32] which originate from bacterial two-component signalling were neither considered in EKPD nor here. In total we included 940 kinases with eukaryotic kinase domain in the analysis, out of which only 553 kinases have yet been functionally characterized, and for 298 out of these kinases we have some regulatory information [15]. Based on the phylogeny, we were able to newly annotate 77 soluble kinases and 108 receptor like kinases (Additional file 1) and assign them to an existing subfamily. Thus, the phylogeny in itself provided an important contribution in definition and classification of protein kinases with unknown function.

### Determination of syntenic regions and family based enrichment

Due to the complex history of duplication events especially in plants, the identification of syntenic regions within and between genomes is a nontrivial task, and conclusions drawn from publicly available datasets may underlie controversial assumptions related to the particular organism under study. To aid such analyses, several recent efforts were made to automate and generalize the process of detecting and evaluating syntenic genome regions. In addition, integrative web-based resources, for example the comparative genomic system platform (CoGe) [5], provide interactive frameworks for query and visualization of syntenic regions within and between genomes. Thus, we focused on the recently published MCScanX utility [7] and based our analysis on the comparison of *Arabidopsis thaliana* against itself, as well as comparisons of close (*Arabidopsis lyrata*) and distant (poplar) relatives.

To determine enrichments for specific types of duplication events in different kinase families, a ratio-based approach (observed/expected) and an enrichment analysis was used. The ratio-based approach showed that tandem duplication ratios tended to be considerably higher than segmental duplication ratios. However, since the detection of subfamily characteristics depends on relative differences between subfamilies, this only marginally affected the conclusions drawn from the analysis. It is important to note that the ratio-based analysis is in general not redundant to the enrichment analysis, since enrichment analysis incorporates the family size parameter. For example, the LRR_clade_3 shows only minor deviations from the median ratios in the boxplot but is found significantly enriched for tandem duplicates (p < 0.001) by Fisher’s exact test and Chi-square residuals.

An interesting observation was that the kinases analyzed here constituted 3.4% of the total genome, but made up 4.5% of all duplicated genes suggesting higher frequencies of duplicated genes in that gene family. This is in line with the observation that kinases are significantly under-represented among single-copy genes [55]. Thus, duplications among kinases showed a tendency to be retained and gave rise to functional diversification.

### Functional diversification and conservation

**Figure 6:**
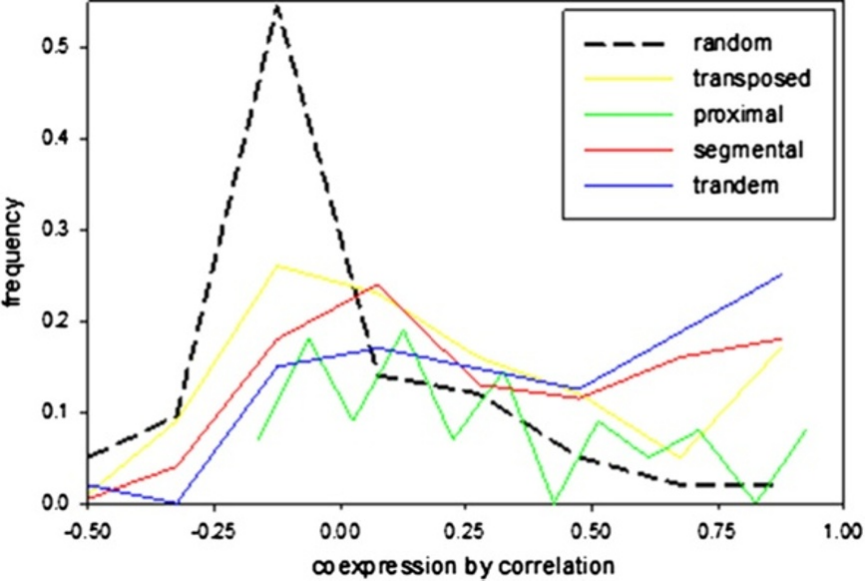
**Frequencies of correlation values for kinase gene pairs.** Colored lines indicate different types of duplications in comparison to randomly sampled kinase-specific gene pairs.

As expected, the coexpression analysis revealed a tendency for segmental duplicates to remain in the same expression context as the ancestral gene, while tandem duplications showed a higher tendency for divergent expression patterns. To put this into a more general context, the distribution of coexpression values for duplication pairs was correlated over all expression sets and plotted for all gene pairs encoding for kinases originating from WGD/segmental, tandem, proximal, transposed duplications and random gene pairs (Figure 6). Random pairs of genes encoding for kinases showed highest frequency of correlation values around zero, while distributions of coexpression correlation for pairs originating from different duplication events were found to be rather flat to bimodal, lacking such a peak. Kinases with segmental and tandem duplications showed maximum correlation values for coexpression. Among genes with proximal duplications we found high frequencies for uncorrelated kinase gene expressions. This confirmed our observation of the tendency for conserved expression patterns within kinase families with high frequencies of segmental duplications (Figure 4). Our findings are also in line with previous studies [7] where several duplication mechanisms were ranked by their potential of introducing functionally divergent duplicates in *Arabidopsis*. There, transposed duplications revealed highest diversification followed by dispersed duplications, tandem duplications and considered segmental duplications as the mechanism with most conserved functions. Thus, clusters of coexpressed genes and phylogenetically related genes preferentially result from segmental duplications. Within the kinases, transposed duplications also lead to a maximum of uncorrelated gene expressions (Figure 6). However, some members of the RLCK 9 subfamily pose a deviation from these global patterns, suggesting a finer grained analysis of subfamilies to be worthwhile. A complete overview of the different duplication events in the kinase families is presented in Additional file 8. The genomic sequences of these kinases would offer additional information such as exon-intron structure and the possibility of testing hypotheses in terms of selective pressures and evolutionary rates, which was beyond the scope of this work.

A tendency for frequent dispersed duplications and associated diversification in gene expression was observed particularly for some of the receptor kinase families. This large kinase subclade was also found to be especially affected by strong diversification through single nucleotide polymorphisms [61, 62]. Since receptor kinases have roles in pathogen defense, self-incompatibility and various developmental processes [63], functional diversification of this gene family allows rapid adaptations to specific environmental conditions. In contrast, some of the soluble kinase families, such as RLCK, MAP-Kinase and SnRK families showed a tendency to be duplicated as a result of segmental duplications and associated functional conservation based on gene coexpression. This is in line with findings that cytoplasmic proteins and proteins involved in cellular metabolism are also less frequently affected by phospho-specific nucleotide polymorphisms [62].

## Conclusions

The prediction and analysis of syntenic blocks and duplication events within gene families of interest can be used to link knowledge from functional biology and proteomics to insights from an evolutionary viewpoint. In our study, the kinome of *Arabidopsis thaliana* was analysed with respect to characteristic patterns of various types of gene duplication modes in combination with subcellular localization, gene expression, and phenotypic data. Summarizing the findings, cytosolic protein kinases and receptor-like protein kinases exhibit different frequencies in the retention of genes duplicated through segmental and tandem duplications, respectively and resulted in different degrees of functional diversification. The phylogeny allowed classification and annotation of yet uncharacterized kinases. The approach undertaken here can be applied to any gene family in any organism with an annotated genome.

### Availability of supporting data

Supplementary material is available as additional files through BioMed Central. The original tree file of the phylogeny has been submitted to Dryad (http://datadryad.org) and is available under the reference number doi: 10.5061/dryad.pq7d7.

## Electronic supplementary material

Additional file 1: **(A)** **List of all 940 kinases and 111 other kinases subjected to this study with respective information about subcellular** **location and proposed annotation based on the phylogeny.** New identified members of a soluble family are marked with one asterisk; re-organization receptor kinases from functional MAPMAN bins into family categorized bins is marked with two asterisks. Family names from EKDP are included as reference. **(B)** Full family names, information on family annotation for soluble kinases as well as extended MAPMAN bins and proposed bin notation of new kinase-related bins. (XLSX 167 KB)

Additional file 2: **Sequence coverage distribution of the analysed kinase domains. Sequences with less than 70% coverage were excluded.** (JPEG 684 KB)

Additional file 3: **Examples for annotation of kinases.** **(A)** Phylogeny of Ste-like MAP3 kinases **(B)** Phylogeny of BRI1-containing leucine-rich repeat kinase family LRR_10. (TIFF 253 KB)

Additional file 4: **Summary of observed (obs) and expected (exp) counts and ratios of segmental (seg) and tandem (tan) duplications.** The classifications (class) correspond to positions of families in the box plot (Figure 3B) with 1 SD and 2 SD indicating one and two standard deviations from the median, respectively. Signs and inequalities indicate the direction of deviation, while families within 1 SD above or below the median were assigned to the ’box’ class. (DOCX 24 KB)

Additional file 5: **Comparison of expression heat maps in combination with phylogenetic relationships.** **(A)** Kinase genes in rows were clustered according to their similarity in several tissue specific gene expression sets (left) and conditional coexpression data (right). **(B)** The kinase genes in rows were reordered according to their phylogenetic distance (branch lengths). Green color indicates strong coexpression, red color indicates high degree of dissimilarity. Coexpression was estimated by Pearson correlation across all expression sets. (TIFF 14 MB)

Additional file 6: **Phenotype information obtained from [** **24] on** ***Arabidopsis*** **kinases.** **(A)** Percentage distribution of phenotype categories for loss-of-function mutations in 76 affected kinases. **(B)** Kinases with described phenotype mapped on the phylogeny. (TIFF 465 KB)

Additional file 7: **List of additional** ***Arabidopsis*** **protein kinases from EKPD and List of two protein kinases analysed here not listed in EKPD [59].** (XLSX 19 KB)

Additional file 8: **Overview of the duplication events in the all of the kinase genes.** Color indicates the duplication mechanism: proximal duplication (green), segmental duplications (red) and transpositions (orange). (PDF 7 MB)
