# Supplementary figures and images for: The Arabidopsis Kinome: phylogeny and evolutionary insights into functional diversification
Source: BMC Genomics. 2014 Jul 1;15(1):548. doi: 10.1186/1471-2164-15-548 (PMC4112214; doi:10.1186/1471-2164-15-548)

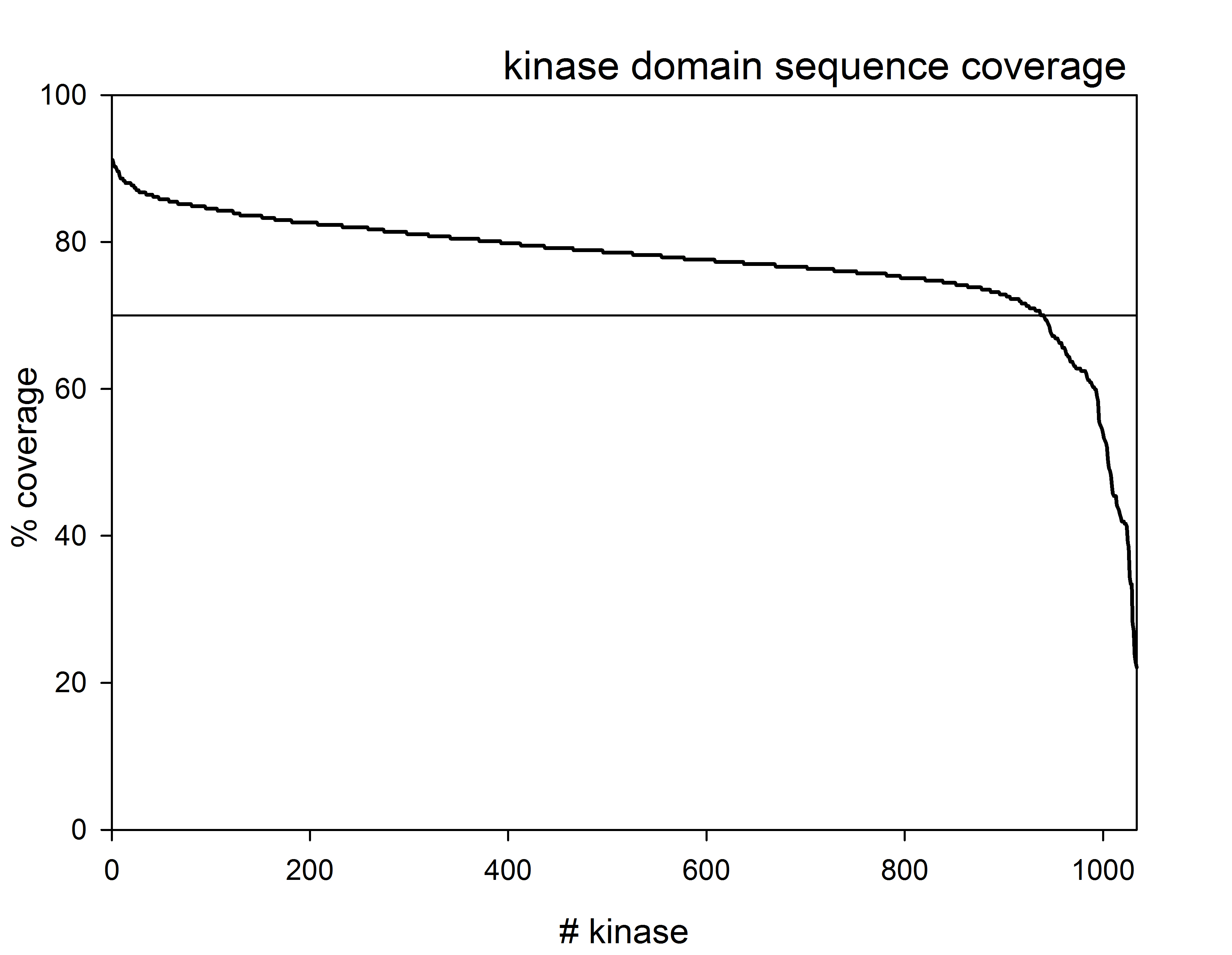

Supplement: Supplementary file 2 — Additional file 2: Sequence coverage distribution of the analysed kinase domains. Sequences with less than 70% coverage were excluded. (JPEG 684 KB) [file 12864_2014_6281_MOESM2_ESM.jpeg]

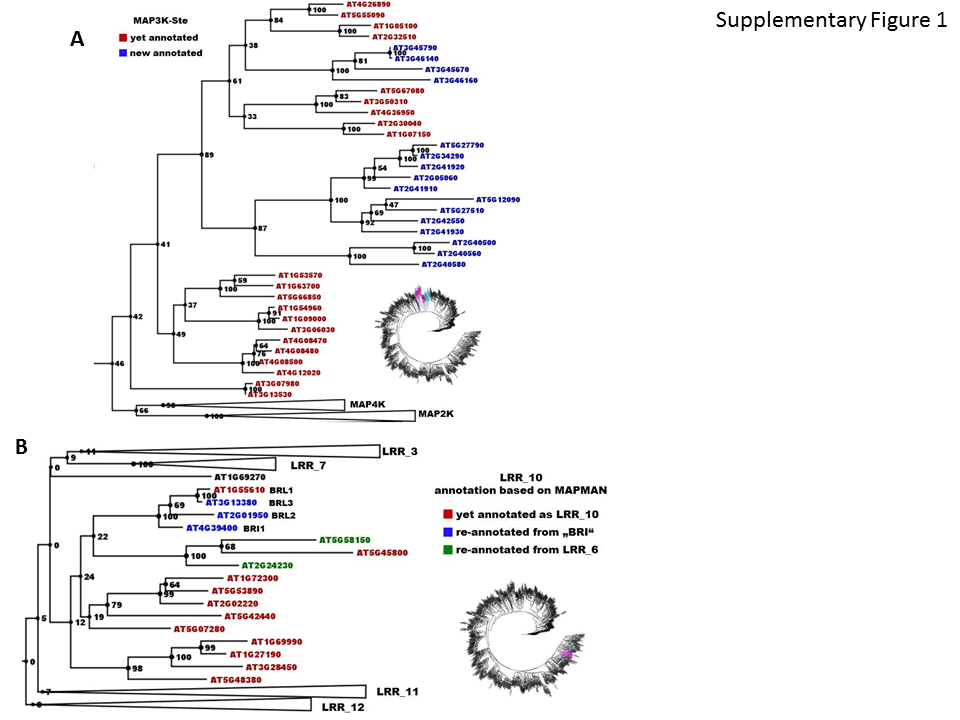

Supplement: Supplementary file 3 — Additional file 3: Examples for annotation of kinases. (A) Phylogeny of Ste-like MAP3 kinases (B) Phylogeny of BRI1-containing leucine-rich repeat kinase family LRR_10. (TIFF 253 KB) [file 12864_2014_6281_MOESM3_ESM.tiff]

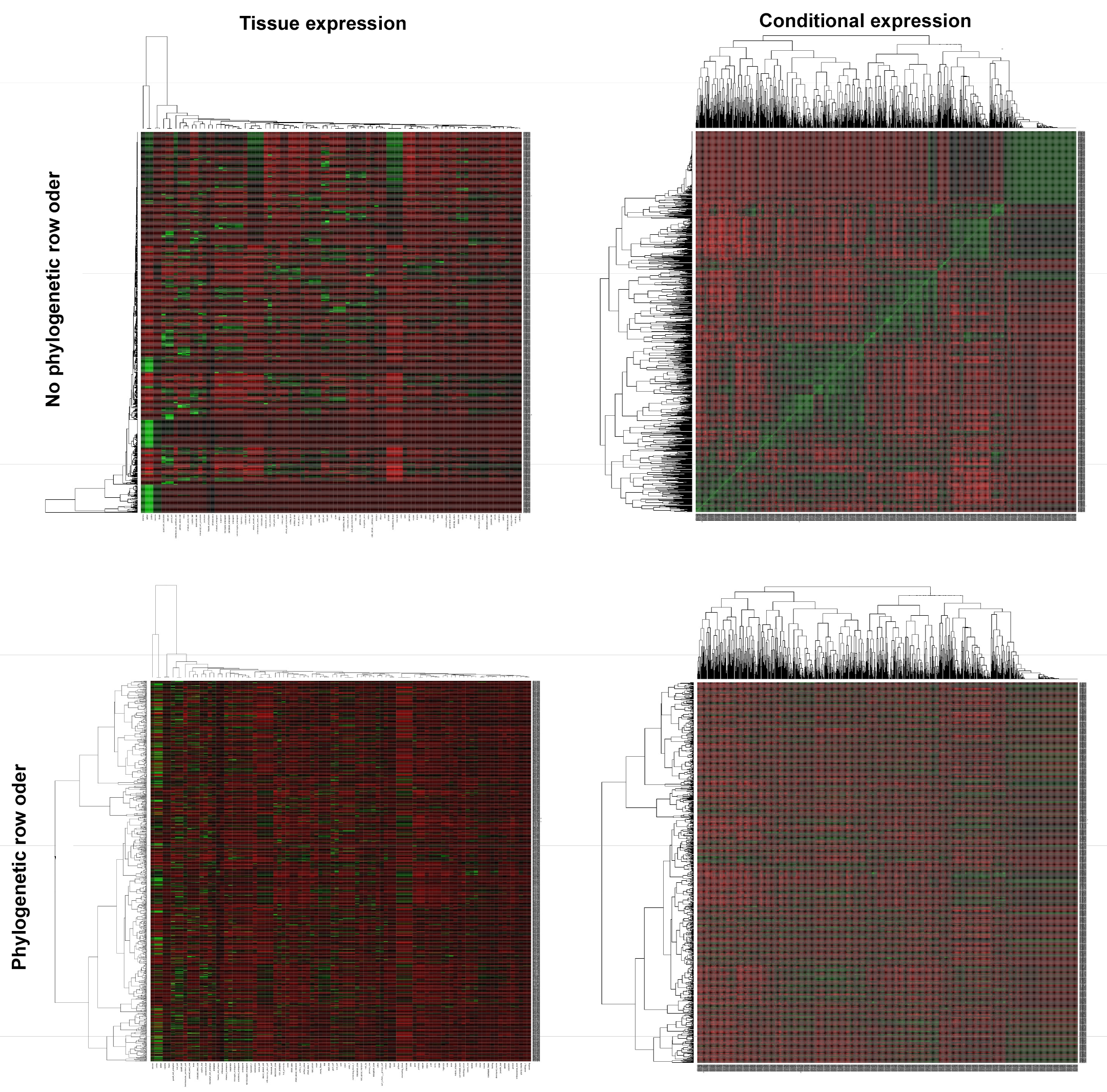

Supplement: Supplementary file 5 — Additional file 5: Comparison of expression heat maps in combination with phylogenetic relationships. (A) Kinase genes in rows were clustered according to their similarity in several tissue specific gene expression sets (left) and conditional coexpression data (right). (B) The kinase genes in rows were reordered according to their phylogenetic distance (branch lengths). Green color indicates strong coexpression, red color indicates high degree of dissimilarity. Coexpression was estimated by Pearson correlation across all expression sets. (TIFF 14 MB) [file 12864_2014_6281_MOESM5_ESM.tiff]

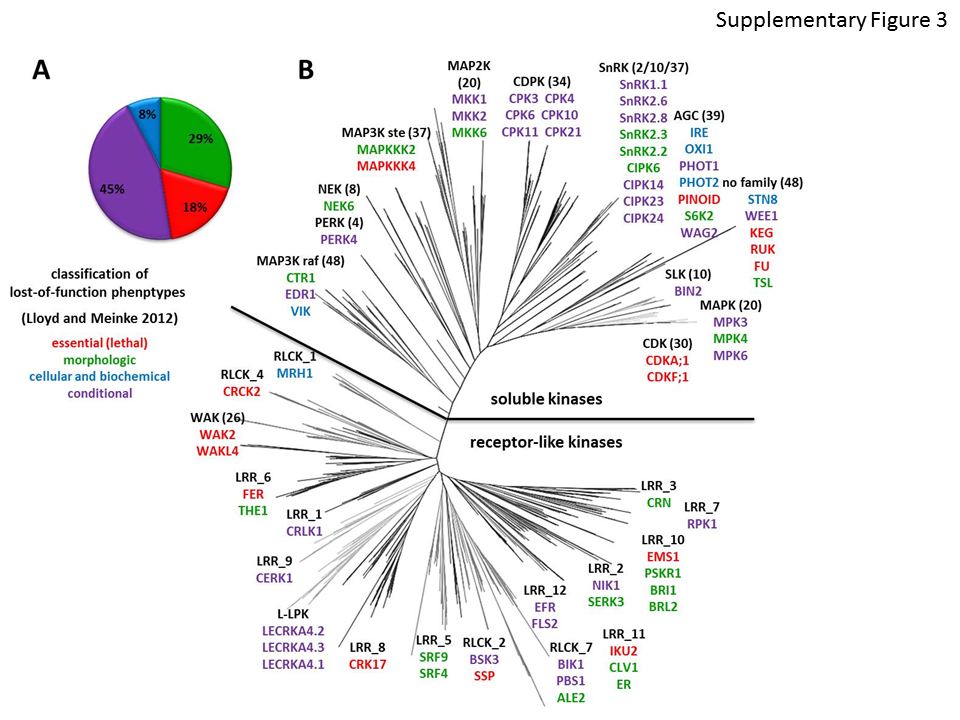

Supplement: Supplementary file 6 — Additional file 6: Phenotype information obtained from [ 24] on Arabidopsis kinases. (A) Percentage distribution of phenotype categories for loss-of-function mutations in 76 affected kinases. (B) Kinases with described phenotype mapped on the phylogeny. (TIFF 465 KB) [file 12864_2014_6281_MOESM6_ESM.tiff]

Segmental Tandem Proviral Transposed Ancestral locus

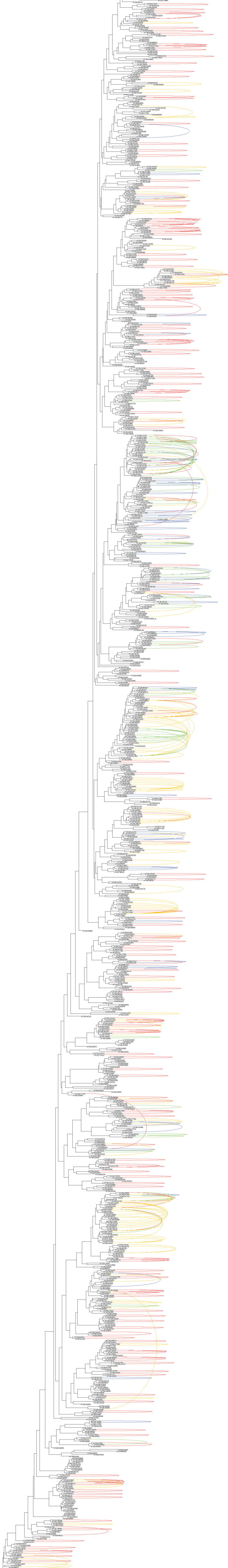

Supplement: Supplementary file 8 — Additional file 8: Overview of the duplication events in the all of the kinase genes. Color indicates the duplication mechanism: proximal duplication (green), segmental duplications (red) and transpositions (orange). (PDF 7 MB) [file 12864_2014_6281_MOESM8_ESM.pdf]
