# Supplementary material for: The Arabidopsis Kinome: phylogeny and evolutionary insights into functional diversification
Source: BMC Genomics. 2014 Jul 1;15(1):548. doi: 10.1186/1471-2164-15-548 (PMC4112214; doi:10.1186/1471-2164-15-548)
Supplement: Supplementary file 4 — Additional file 4: Summary of observed (obs) and expected (exp) counts and ratios of segmental (seg) and tandem (tan) duplications. The classifications (class) correspond to positions of families in the box plot (Figure 3B) with 1 SD and 2 SD indicating one and two standard deviations from the median, respectively. Signs and inequalities indicate the direction of deviation, while families within 1 SD above or below the median were assigned to the ’box’ class. (DOCX 24 KB) [file 12864_2014_6281_MOESM4_ESM.docx]

**Table 2**: Summary of observed (obs) and expected (exp) counts and ratios of segmental (seg) and tandem (tan) duplications The classifications (class) correspond to positions of families in the boxplot (fig.4) with 1SD and 2SD indicating one and two standard deviations from the median, respectively. Signs and inequalities indicate the direction of deviation, while families lying within 1SD above or below the median are assigned to the ’box’ class.

|  | family | seg obs | seg exp | seg ratio | seg class | tan obs | tan exp | tan ratio | tan class |
| --- | --- | --- | --- | --- | --- | --- | --- | --- | --- |
| 1 | AGC | 18 | 29.25 | 0.62 | box | 2 | 1.51 | 1.32 | box |
| 2 | AURORA | 0 | 2.25 | 0.00 | *<* -1SD | 0 | 0.07 | 0.00 | box |
| 3 | CDK | 17 | 22.50 | 0.76 | box | 0 | 0.99 | 0.00 | box |
| 4 | CDPK | 9 | 25.50 | 0.35 | box | 1 | 1.51 | 0.66 | box |
| 5 | CK II | 3 | 3.00 | 1.00 | *>* +1SD | 1 | 0.07 | 13.51 | *>* +2SD |
| 6 | CKL | 9 | 9.75 | 0.92 | *>* +1SD | 0 | 0.35 | 0.00 | box |
| 7 | CPKRK | 0 | 6.00 | 0.00 | *<* -1SD | 0 | 0.07 | 0.00 | box |
| 8 | LRR 11 | 10 | 28.50 | 0.35 | box | 2 | 1.51 | 1.32 | box |
| 9 | LRR 12 | 0 | 6.00 | 0.00 | *<* -1SD | 2 | 0.07 | 27.03 | *>* +2SD |
| 10 | LRR 14 | 0 | 1.50 | 0.00 | *<* -1SD | 0 | 0.07 | 0.00 | box |
| 11 | LRR 2 | 6 | 10.50 | 0.57 | box | 1 | 0.35 | 2.89 | box |
| 12 | LRR 5 | 2 | 7.50 | 0.27 | box | 0 | 0.07 | 0.00 | box |
| 13 | LRR clade 1 | 14 | 53.25 | 0.26 | box | 5 | 6.19 | 0.81 | box |
| 14 | LRR clade 2 | 4 | 8.25 | 0.48 | box | 0 | 0.35 | 0.00 | box |
| 15 | LRR clade 3 | 19 | 59.25 | 0.32 | box | 30 | 6.19 | 4.84 | box |
| 16 | LRR clade 4 | 34 | 51.00 | 0.67 | box | 0 | 4.65 | 0.00 | box |
| 17 | L LPK | 9 | 28.50 | 0.32 | box | 11 | 1.51 | 7.28 | *>* +1SD |
| 18 | MAP2K | 6 | 7.50 | 0.80 | *>* +1SD | 0 | 0.07 | 0.00 | box |
| 19 | MAP3K | 34 | 63.75 | 0.53 | box | 7 | 7.76 | 0.90 | box |
| 20 | MAPK | 10 | 15.00 | 0.67 | box | 0 | 0.35 | 0.00 | box |
| 21 | Mixed clade 1 | 8 | 41.25 | 0.19 | box | 11 | 3.33 | 3.30 | box |
| 22 | NEK | 2 | 5.25 | 0.38 | box | 0 | 0.07 | 0.00 | box |
| 23 | PKRK | 0 | 3.00 | 0.00 | *<* -1SD | 0 | 0.07 | 0.00 | box |
| 24 | RK 1 | 2 | 15.00 | 0.13 | *<* -1SD | 5 | 0.35 | 14.45 | *>* +2SD |
| 25 | RLCK 10 | 6 | 7.50 | 0.80 | *>* +1SD | 0 | 0.07 | 0.00 | box |
| 26 | RLCK 10A | 2 | 2.25 | 0.89 | *>* +1SD | 0 | 0.07 | 0.00 | box |
| 27 | RLCK 9 | 7 | 14.25 | 0.49 | box | 2 | 0.35 | 5.78 | *>* +1SD |
| 28 | RLCK clade 1 | 18 | 30.00 | 0.60 | box | 0 | 1.51 | 0.00 | box |
| 29 | RLCK clade 2 | 40 | 54.00 | 0.74 | box | 3 | 6.19 | 0.48 | box |
| 30 | SLK | 0 | 7.50 | 0.00 | *<* -1SD | 0 | 0.07 | 0.00 | box |
| 31 | SnAK | 0 | 1.50 | 0.00 | *<* -1SD | 0 | 0.07 | 0.00 | box |
| 32 | SnRK1 | 2 | 2.25 | 0.89 | *>* +1SD | 0 | 0.07 | 0.00 | box |
| 33 | SnRK2 | 6 | 7.50 | 0.80 | *>* +1SD | 0 | 0.07 | 0.00 | box |
| 34 | SnRK3 | 13 | 20.25 | 0.64 | box | 2 | 0.99 | 2.03 | box |
| 35 | WNK | 4 | 8.25 | 0.48 | box | 0 | 0.35 | 0.00 | box |
| 36 | rk a | 0 | 2.25 | 0.00 | *<* -1SD | 0 | 0.07 | 0.00 | box |
| 37 | soluble | 21 | 45.75 | 0.46 | box | 4 | 4.65 | 0.86 | box |
